# Nitric oxide releasing graphene for next-generation therapeutics☆

**DOI:** 10.1016/j.addr.2025.115676

**Published:** 2025-08-18

**Authors:** Tanveer A. Tabish, Craig A. Lygate

**Affiliations:** a Division of Cardiovascular Medicine, Radcliffe Department of Medicine, British Heart Foundation (BHF) Centre of Research Excellence, University of Oxford, Oxford OX3 7BN, United Kingdom; b School of Cardiovascular and Metabolic Health, College of Medical, Veterinary and Life Sciences, University of Glasgow, Glasgow G12 8TA, United Kingdom

## Abstract

Nitric oxide (NO) is a powerful signalling molecule and plays a central role in numerous physiological processes, most notably, in the cardiovascular, immune and central nervous systems. While organic nitrates, exemplified by nitroglycerin, have been used for over a century to deliver therapeutic NO, the search for novel drugs capable of selectively increasing NO bioavailability has continued unabated. Delivery of NO is hindered by its gaseous nature, extreme reactivity, short half-life and potential for systemic toxicity. To address these challenges, controlled NO delivery systems are highly desirable, offering precise release at the site of action over defined periods. Recent advances have focused on nanoparticles for injectable or implantable use, enabling sustained, targeted NO release while degrading safely. Among these, graphene nanostructures have emerged as efficient NO carriers, since they can be specifically designed to deliver NO gas or donor compounds due to their tunable surface chemistry, easy chemical modification and good biocompatibility. In this review, we discuss the latest developments in NO-releasing graphene formulations, alongside key applications in cardiovascular diseases, antimicrobial therapy and cancer treatment.

## Graphical abstract

**Graphic Alabstract:**
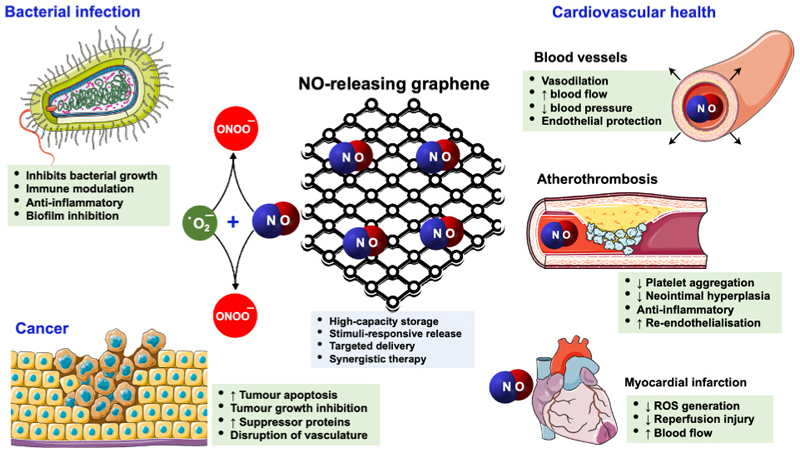

## 1 Introduction

### 1.1 Historical perspective

Nitric oxide (NO) is a gaseous molecule with primordial origins, hypothesized as a contributor to the early processes leading to the origin of life on Earth [1]. In the prehuman world, the production of NO by lightning in an anoxic atmosphere, may have driven the evolution of key biochemical pathways, including respiration and the nitrogen cycle [2,3]. Millennia later, NO was rediscovered as a key biological signaling molecule in blood vessels, identified by Furchgott and Ignarro as endothelium-derived relaxing factor (EDRF), while Murad demonstrated that nitroglycerin releases NO to mediate its vasodilatory effects [2]. These ground-breaking discoveries dramatically altered the course of vascular biology and inaugurated extensive research on the key roles of NO in many physiological processes, earning the Nobel Prize in Physiology or Medicine for the three scientists in 1998 [4]. NO has since been shown to play a variety of roles in mammalian biology, notably regulating vasodilation, neurotransmission, host immunity and inflammation.

This review will provide a brief introduction to the physiology and biochemistry of endogenous NO and the current therapeutic options for increasing NO bioavailability. The properties and rational design of graphene as a NO-delivery system will be explored and illustrated by examples of preclinical development from the fields of cardiovascular disease, antimicrobial and anti-cancer therapies.

### 1.2 Biochemistry of endogenous NO production

The human body produces a significant amount of NO from the enzymatic conversion of L-arginine and molecular oxygen to NO and L-citrulline via three distinct isoforms of NO synthase (NOS), namely, neuronal (nNOS or NOS1), inducible (iNOS or NOS2) and endothelial (eNOS or NOS3) [5]. nNOS and eNOS are constitutively expressed enzymes that are dependent on intracellular calcium levels and produce relatively low quantities of NO [5]. iNOS is a calcium-independent enzyme that produces large quantities of NO and is induced by proin-flammatory cytokines, endotoxins and neuropeptides. NOS is a functional dimer, with each monomer comprising two domains: an N-terminal oxygenase domain and a C-terminal reductase domain. The oxygenase domain contains a cytochrome P450 type heme centre and a binding site for the essential cofactor tetrahydrobiopterin (BH4) [6]. The reductase domain contains nicotinamide adenine dinucleotide phosphate hydrogen (NADPH), flavin adenine dinucleotide (FAD) and flavin mononucleotide (FMN) binding sites. Binding of the calcium-binding regulatory protein calmodulin (CaM) facilitates the electron transfer from the reductase domain to the heme group in the oxygenase domain, which is essential for O_2_ to bind to NOS heme, a key step in NO production [7].

### 1.3 Canonical NO signalling in blood vessels

**Fig. 1:**
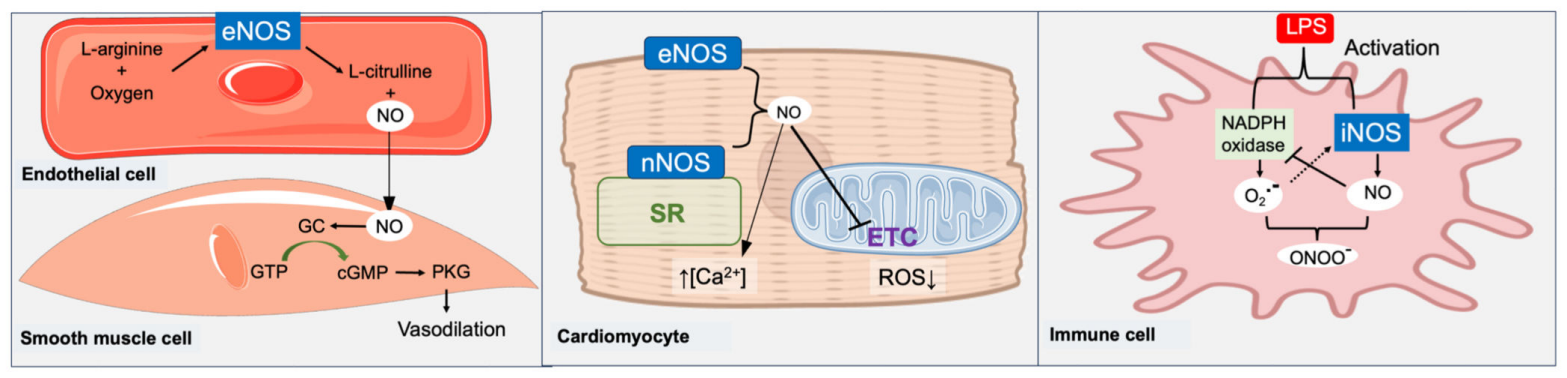
Mechanisms of nitric oxide (NO) signaling in cardiovascular physiology, infection and cancer. NO is produced from L-arginine and molecular oxygen by NO synthase (NOS) enzymes, which include endothelial (eNOS), neuronal (nNOS) and inducible (iNOS) isoforms. In endothelial cells, eNOS-derived NO diffuses to smooth muscle cells (SMCs) where it activates soluble guanylate cyclase (sGC), which converts guanosine triphosphate (GTP) to the secondary messenger, cyclic guanosine monophosphate (cGMP). cGMP then activates protein kinase G (PKG), which phosphorylates multiple downstream proteins resulting in SMC relaxation and vasodilation. Activating this pathway in endothelial cells is important after vascular injury since it promotes re-endothelialization and angiogenesis, while preventing unwanted neointimal hyperplasia by inhibiting SMC proliferation and migration. In cardiomyocytes, nNOS is located at the sarcoplasmic reticulum (SR), where NO regulates calcium signaling to fine tune cardiac contraction and relaxation. NO also reduces reactive oxygen species (ROS) generation by inhibiting xanthine oxidoreductase and mitochondrial respiration at electron transport chain (ETC) complexes I and IV, thereby protecting against Ca^2+^ overload and oxidative stress. Endogenous cytokines or lipopolysaccharide (LPS) from bacteria can activate immune cells (such as macrophages) and induce iNOS expression to produce high levels of NO. They also activate nicotinamide adenine dinucleotide phosphate oxidase (NADPH oxidase) to generate superoxide $\left({\text{O}}_{2}^{-}\right)$, which reacts with NO to form peroxynitrite (ONOO ^-^), an oxidant which induces tumor cell apoptosis and exhibits antibacterial effects by disrupting microbial iron-sulfur (Fe-S) proteins and DNA.

The vascular system could be heralded as ‘the archetypal paradigm’, since the discovery of NO is rooted in studies of the vascular endothelium. The endothelium is a monolayer of endothelial cells (ECs) which constitutes the inner lining of blood vessels (arteries, veins and capillaries) as well as the lymphatic system. NO is continuously produced by eNOS in ECs (Fig. 1), where it diffuses into adjacent vascular smooth muscle cells to activate soluble guanylate cyclase (sGC), leading to increased levels of the second messenger, cyclic guanosine monophosphate (cGMP) and activation of protein kinase G (PKG). PKG phosphorylates downstream proteins resulting in smooth muscle cell relaxation and vasodilation, while the same pathway in platelets and white blood cells (e.g. leukocytes) acts to inhibit adhesion and subsequent activation, thereby preventing platelet aggregation and leukocyte-endothelial interactions [8,9]. In this way, NO plays a central role in maintaining healthy vascular homeostasis by reducing blood pressure and preventing unnecessary blood clotting and inflammatory processes. NO also acts as a neurotransmitter and facilitates penile erection through its neurotransmitter and local vasodilatory effects, with insufficient production of NO contributing to impotence [24].

### 1.4 NO in the heart

Neuronal NOS is the primary source of NO within cardiac muscle cells, where it is bound to the sarcoplasmic reticulum to modulate intracellular calcium handling, and thereby regulate cardiac relaxation and contraction [10,11]. NO also maintains nitroso-redox balance by suppressing activity of xanthine oxidoreductase, which is one of the major sources of superoxide anions in the heart [12]. Furthermore, it has a signalling role via *S*-nitrosylation of proteins including inhibition of the mitochondrial respiratory chain, which may have utility in the prevention of ischaemia–reperfusion injury [13].

### 1.5 NO in pathogen defence

As outlined above, low levels of NO from the endothelium has an anti-inflammatory effect by maintaining immune cells in a quiescent state. However, when immune cells such as macrophages are activated (e.g. by cytokines or bacterial endotoxins), they can produce large quantities of NO via iNOS, which plays a critically important role in host defence by modulating immune responses and killing pathogens. NO itself does not directly induce antimicrobial effects, it reacts with oxygen and superoxide to produce a variety of antimicrobial reactive nitrogen species including peroxynitrite (OONO^–^), *S*-nitrosothiols (RSNO), nitrogen dioxide (NO_2_), dinitrogen trioxide and dinitrosyl-iron complexes [14,15]. These reactive intermediates cause oxidative and nitrosative stress by modifying DNA, disrupting enzyme activity and damaging bacterial membranes through lipid peroxidation. Among these, peroxynitrite demonstrates significantly higher cytotoxicity than either NO or superoxide alone [16] and has the ability to traverse cell membranes [14].

### 1.6 NO in cancer

NO plays multifaceted and sometimes opposing roles in the setting of cancer, with effects largely dependent on concentration, cellular localisation and the tumor microenvironment [17]. NO produced by activated macrophages can be cytotoxic to cancer cells via the same mechanisms as for pathogens [18], with concentrations ranging from 1 μM to 1 mM [19] resulting in cell death through necrosis, whereas concentrations between 10 nM −1 μM may contribute to anti-apoptotic processes [23]. Cancer cells are particularly sensitive to the toxic effects of NO due to impaired redox homeostasis and elevated ROS levels, which compromises their capacity to resist oxidative and nitrosative stress. This results in disruption of mitochondrial function (and thereby energy production), DNA damage, and up-regulation of cell death pathways, e.g. p53 activation and opening of the mitochondrial permeability transition pore. Collectively, these result in cell cycle arrest, inhibition of angiogenesis and tumor cytotoxicity (Fig. 1) [20]. NO also has immunomodulatory properties, acting to stimulate cytotoxic T lymphocytes and inhibit immunosuppressive tumor-associated macrophages [21]. The flip-side is that iNOS can be expressed in tumor cells and this is associated with tumor progression and aggressive tumor phenotypes [22]. The tumor microenvironment (comprising tumor, immune and stromal cells), is also an important factor, which poses significant challenges to the therapeutic delivery of NO [23,24]. For example, hypoxic regions may restrict the diffusion and reactivity of NO, thereby limiting responses. Furthermore, the concentration-dependent effects of NO acts as a double edged sword, since low levels can promote angiogenesis and thereby tumor growth, whereas high concentrations are cytotoxic for cancer cells. This concentration gradient provides opportunities for controlled and targeted delivery of NO.

## 2 Therapeutic delivery of NO

### 2.1 The physicochemical limitations of NO

The clinical use of NO-releasing compounds in the form of organic nitrates and nitrites or direct NO gas inhalation represents a key therapeutic option for various pathologies. NO is both lipophilic and hydrophilic, with a small Stokes radius that facilitates its easy passage across cellular membranes. However, as a radical gas, NO is inherently unstable and rapidly oxidizes to toxic NO by-products in the presence of oxygen (e.g. peroxynitrite or nitrogen dioxide). Due to its short half-life (t_1/2_ ≤ 1 s) and short diffusion distance (up to 200 μm), its biological effects are confined to its site of production [25]. These intrinsic physicochemical properties significantly limit therapeutic use of NO gas, while limitations of NO donor drugs include chemical instability, tissue penetration, development of tolerance and adverse effects such as headache [26].

### 2.2 Clinical use of NO-based therapies

Reduced NO bioavailability is a hallmark of many pathologies, particularly diseases of the cardiovascular system [11]. Current strategies to correct this deficit span three distinct categories: direct NO gas; nitric oxide donors (e.g. organic nitrates as pro-drugs); and drugs that amplify downstream signaling pathways. Each category utilizes distinct features of NO to address various disease states. Inhaled NO gas has been an FDA-approved treatment since 1999 for hypoxic respiratory failure and pulmonary hypertension in neonates [27]. The short diffusion distance is an advantage in this case, since NO works to cause local pulmonary vasodilatation and bronchodilation, while reducing inflammation and cell proliferation.

Pharmacological NO donors have been a mainstay in medicine for more than a century. The earliest class of NO donors are organic nitrates (e.g. amyl nitrite, nitroglycerin a.k.a. glyceryl trinitrate, and isosorbide mononitrate), which are classic vasodilators used clinically to treat angina, heart failure and pulmonary hypertension [28]. Nitroglycerin is commonly prescribed for acute treatment of angina and releases 1 mol equivalent of NO following bioconversion by the enzyme mitochondrial aldehyde dehydrogenase (mtALDH) [29]. Nitrate tolerance (tachyphylaxis) is a common consequence of prolonged nitrate use and results in a gradual loss of therapeutic efficacy. This is driven by impaired enzymatic NO production, desensitization of the sGC signaling pathway, oxidative stress leading to NO depletion and neurohormonal counterregulation (e.g. increased sympathetic activity) [30,31]. Other clinical applications of NO donors include an NO-releasing lozenge for lowering blood pressure in prehypertensive patients by triggering nitrate-based NO release [29], sodium nitroprusside for acute management of hypertension [32] and latanoprostene bunod to lower intraocular pressure for the treatment of glaucoma [33].

Clinically approved drugs which improve NO signaling or target its downstream pathways include phosphodiesterase inhibitors such as sildenafil (Viagra), which inhibits breakdown of cGMP to amplify NO signaling [34] and is used principally for erectile dysfunction, but also for pulmonary hypertension. Similarly, sGC stimulators such as Vericiguat (licensed in the UK for the treatment of heart failure) directly activate sGC, restoring cGMP levels independent of NO, which circumvents the problem of nitrate tolerance [35].

There is evidence to support the repurposing of NO donors as antitumor therapies [36], however there are currently no licensed NO-based therapeutics specifically designed to target cancer or to harness the antimicrobial effects of NO. In part, this reflects the limitations of targeted NO delivery using conventional pro-drug approaches.

### 2.3 Pre-clinical development of NO-releasing compounds

A variety of NO-releasing compounds are currently in development. Typical examples include metal nitrosyl complexes (i.e. complexes of manganese, iron and ruthenium) [37], NONOates (N-dia-zeniumdiolates) that release NO spontaneously under physiological conditions [15], *S*-nitrosothiols, which release NO via thiol-based reactions [38] and nitrooxy derivatives, which incorporate NO-releasing moieties into organic compounds for controlled delivery [15]. Of these compounds, NONOates are the most widely studied and are synthesized by reacting primary or secondary amines with NO under high pressure in the presence of a base. They spontaneously release two equivalents of NO per donor in aqueous environments either through hydrolysis or via thermal, photochemical and enzymatic reactions (catalyzed by esterases). However, a notable limitation of NONOates is their potential to generate carcinogenic nitrosamines as a byproduct of their degradation [39] and for the uncontrolled burst release of NO [40].

*S*-nitrosothiols (RSNOs) are exemplified by the endogenous NO donors *S*-nitrosoglutathione (GSNO) and *S*-nitrosocysteine (SNO-Cys), and by *S*-nitroso-N-acetylpenicillamine (SNAP), which mimic the physiological processes of natural NO transport and release. They utilize various NO release pathways including thermal or photoinduced decomposition and catalytic decomposition with amines, organoselenium or metal ions such as copper and ferrous. Thermal or photoinitiated decomposition cleaves the S–N bond homolytically, producing thiyl (RS˙) and NO radicals, while the thiyl radical subsequently reacts with additional RSNO to form disulfides (RSSR) and further NO [38]. In contrast, catalytic decomposition by amines, metal ions or organoselenium produces NO by lowering the activation energy required to cleave the S–N bond [41]. RSNOs offer significant advantages over other NO donors including low cytotoxicity and slow NO release via transnitrosation. GSNO, a naturally occurring NO substrate in the bloodstream, has been widely studied for a wide range of biomedical applications [38], while the excellent stability of SNAP makes it an ideal NO donor for long-term NO delivery applications such as surface coatings [42]. However, rapid systemic clearance of low molecular weight RSNOs typically necessitates repeated dosing [43].

Further work is required to realize the therapeutic potential of NO donors such as metal nitrosyls, NONOates and RSNOs. Key challenges to be addressed include short half-life, limited bioavailability [44], formation of toxic byproducts (e.g. cyanide, peroxynitrite, nitrosamines) [45], improved targeting to reduce off-target side-effects (such as headache, hypotension or oxidative stress) [15] and instability under various environmental conditions (e.g. light, temperature and pH) [9]. Addressing these challenges has stimulated interest in developing NO-release systems based on nanomaterials in order to achieve targeted and controlled release.

### 2.4 The promise of nanomaterials for NO delivery

**Fig. 2:**
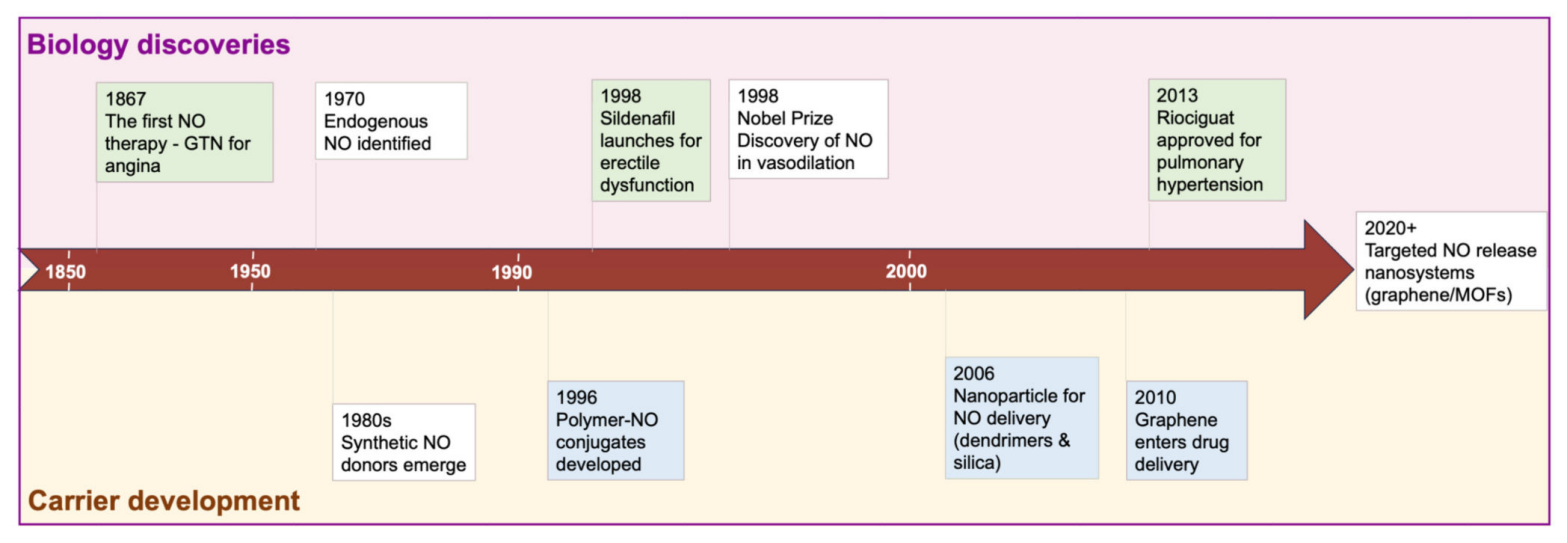
Milestones in nitric oxide (NO) biology and therapeutic carrier development. (Top) Key discoveries in NO biology: from the first clinical use of glyceryl trinitrate (GTN) for angina (1867) to phosphodiesterase-5 (PDE5) inhibitor (sildenafil) and soluble guanylate cyclase (sGC) stimulators (riociguat). (Bottom) NO carrier technologies from small molecule donors to advanced nanomaterials such as graphene and metal-organic frameworks (MOFs). Color indicates development stage: 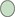 clinical use 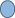; preclinical research; 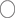 fundamental discovery. Abbreviations: GTN (glyceryl trinitrate); NO (nitric oxide); MOFs (metal-organic frameworks).

Nanomaterials offer the potential to optimize pharmacokinetic and pharmacodynamic properties for the controlled, predictable and targeted release of NO [46]. Examples include liposomes [47], dendrimers [48], silica [49], gold [50] and metal–organic frameworks (MOFs) [51–54]. Nanomaterials offer unique features, including ultra-small size, high surface area-to-volume ratio, hydrolytic degradability, chemical tunability, simple functionalization, controllable drug release kinetics and the ability to target specific cells, subcellular compartments or tissues [55]. Owing to these properties, nanomaterials have been a popular area of research for biomedical applications such as therapeutics and diagnostics, with a number of approaches successfully transitioning to clinical use [56]. Among the various nanomaterials studied for NO delivery, graphene and its derivatives (e.g. graphene oxide) exhibit distinctive and favourable physicochemical characteristics, which will be discussed in the following sections. Fig. 2 illustrates a timeline mapping milestones in NO research, biological discoveries (top) with the parallel evolution of therapeutic delivery systems (bottom).

## 3 Graphene-based materials

Graphene consists of an atomically thick sheet of 2D hexagonal (honeycomb) lattice of sp^2^-hybridized carbon atoms, which can serve as a building block for various other carbon nanomaterials including carbon nanotubes and fullerene. More than 60 years ago, Slonczewski and Weiss theorized graphene, but its practical existence was considered impossible due to thermodynamic challenges highlighted by Landau, Peierls and Mermin [57]. However, in 2004, Novoselov and Geim, isolated single layer graphene from its three-dimensional parent material, graphite, using the scotch tape method, for which they were awarded the Nobel Prize in Physics in 2010 [58]. Since then, graphene has been studied extensively for a wide range of applications including semiconductors, flexible displays, energy, catalysis and healthcare due to its remarkable physicochemical properties. These include a very high surface area (2630 m^2^/g), superior mechanical strength (Young’s modulus of 1100 GPa), excellent thermal/electrical conductivity and optical properties [59,60]. The hydrophobicity of pristine graphene limits its use in living systems, but this can be overcome via the introduction of oxygen-based functional groups onto graphene, thereby producing hydrophilic derivatives such as graphene oxide (GO), GO foam and GO quantum dots. These have been developed for a wide variety of biomedical applications, including drug delivery platforms, light-mediated therapies, diagnostics, bioimaging, biosensing, regenerative medicine and tissue engineering. In the context of NO delivery, the research to date has primarily focused on the development of these GO-based systems.

GO contains sp^2^-hybridized carbons in its aromatic lattice alongside, sp^3^-hybridized carbons with hydroxyl (−OH), epoxide (−O-) and carboxylic acid (COOH) groups at the edges. The presence of these functional groups facilitates interactions with electronegative biomolecules and drugs via hydrogen bonding. The carboxylic acid groups allow electrostatic interactions and can be harnessed for pH-sensitive release of molecules. The existence of the aromatic ring at the plane of GO supports drug or molecule binding via π-π stacking and hydrophobic forces. Additionally, the covalent bonding of GO with drugs and molecules enables stable and precise attachment. The synthesis of GO generally involves the oxidation of graphite using strong acids and oxidizing agent, with Hummer’s method or its modified protocols being the most commonly used. The oxidation process partially disrupts the conjugated π-electron system, resulting in reduced electrical conductivity, but leaves sp^2^-hybridized regions intact. The introduction of oxygen-containing hydrophilic functional groups, provide GO with unique properties, including strong affinity for aromatic rings and good dispersibility in aqueous media, making it highly suitable for biomedical applications [61].

The broader applications of graphene as a drug delivery platform have been extensively reviewed elsewhere [62–65], however the potential of NO-releasing graphene for therapeutic use remains under-developed. Graphene oxide (GO) has particularly attractive properties for NO delivery. Firstly, the presence of surface functional groups allows for the direct physical encapsulation of NO gas or covalent/non-covalent attachment of NO donors. Secondly, the high specific surface area and the presence of defect sites and vacancies, significantly improves NO stability and allows for controlled and favorable release kinetics. Thirdly, it facilitates stimuli-sensitive NO release, triggered by internal or external stimuli such as light or pH, allowing precise and site-specific delivery. These features make GO an ideal candidate for delivering NO, serving as a NO-generating catalyst and as an NO-storage material, which can be formulated as a composite with hydrogels or other materials.

The rest of this review will provide critical analysis of promising graphene-based NO delivery platforms including catalytically generating NO from endogenous NO reservoirs, physical encapsulation of NO gas and covalently bound NO donors. Furthermore, example applications in cardiovascular disease, antibacterial, antibiofilm and cancer are discussed.

## 4 Design and synthesis of NO-releasing graphene

**Fig. 3:**
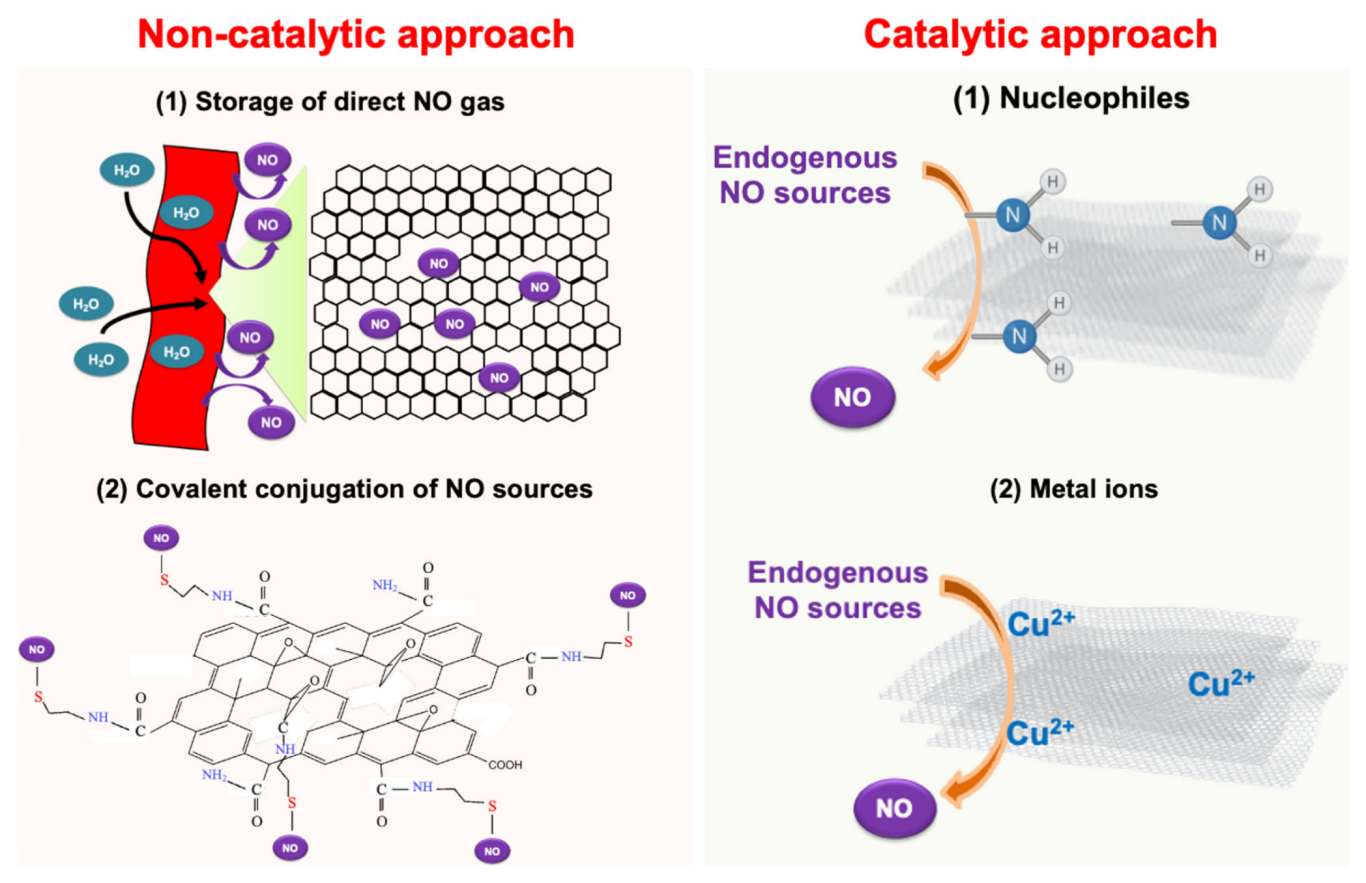
Schematic diagram illustrating NO release and generation mechanisms from graphene-based materials via catalytic and non-catalytic approaches. Non-catalytic NO delivery occurs through: (1) physical incorporation of direct NO gas into graphene pores and (2) covalent conjugation of NO donors onto graphene surfaces via thiol or other functional groups (Figure modified from reference [66]). Catalytic NO generation involves the decomposition of endogenous NO sources, such as *S*-nitrosothiols (RSNOs), facilitated by: (1) nucleophilic attack and (2) metal ion-mediated cleavage (e.g., Cu^2+^ ions) of S–NO bonds, releasing bioactive NO.

The strategies for NO delivery from graphene can be broadly classified into two main categories: (1) catalytic generation of NO from endogenous *S*-nitrosothiols using functionalized graphene as the catalyst; and (2) the non-catalytic approach involving release of stored NO, which can be in the form of physically adsorbed NO gas or the chemical encapsulation of NO donors, that release NO upon physiologically or externally applied stimuli. Both approaches are outlined schematically in Fig. 3 and representative studies highlighting each of these strategies are discussed in Section 5 and summarized in Table 1.

### 4.1 Catalytic generation of NO

Endogenous NO substrates such as RSNO and GSNO can decompose to generate NO in the presence of catalysts via multiple mechanisms, as follows: (1) amine or thiol-containing compounds (e.g. polyethyleneimine (PEI) [67] or L-cysteine [68]) can act as nucleophiles and attack electrophilic SNO groups to generate NO. (2) Transition metalions derived from Cu^2+^, Hg^2+^, Fe^2+^, Ag^+^ or tellurium [69], e.g. Cu (II) is reduced by trace amounts of thiolate to form Cu(I), which then interacts with RSNOs to generate NO and regenerate thiolate and Cu (II). (3) The selenium-containing enzyme glutathione peroxidase (GPx), which is present in blood or can be incorporated into compounds, can catalytically decompose RSNO into NO in the presence of glutathione (GSH) as a reducing substrate [70]. (4) Enzymatic release, such as via protein disulfide isomerase or superoxide dismutase [71]. The potential advantage of NO-generating materials is that sustained NO delivery can be achieved because endogenous RSNO in circulating blood is continuously replenished. Such an approach has potential applications for long-term NO release for medical devices including vascular stents, bypass grafts and catheters.

**Fig. 4:**
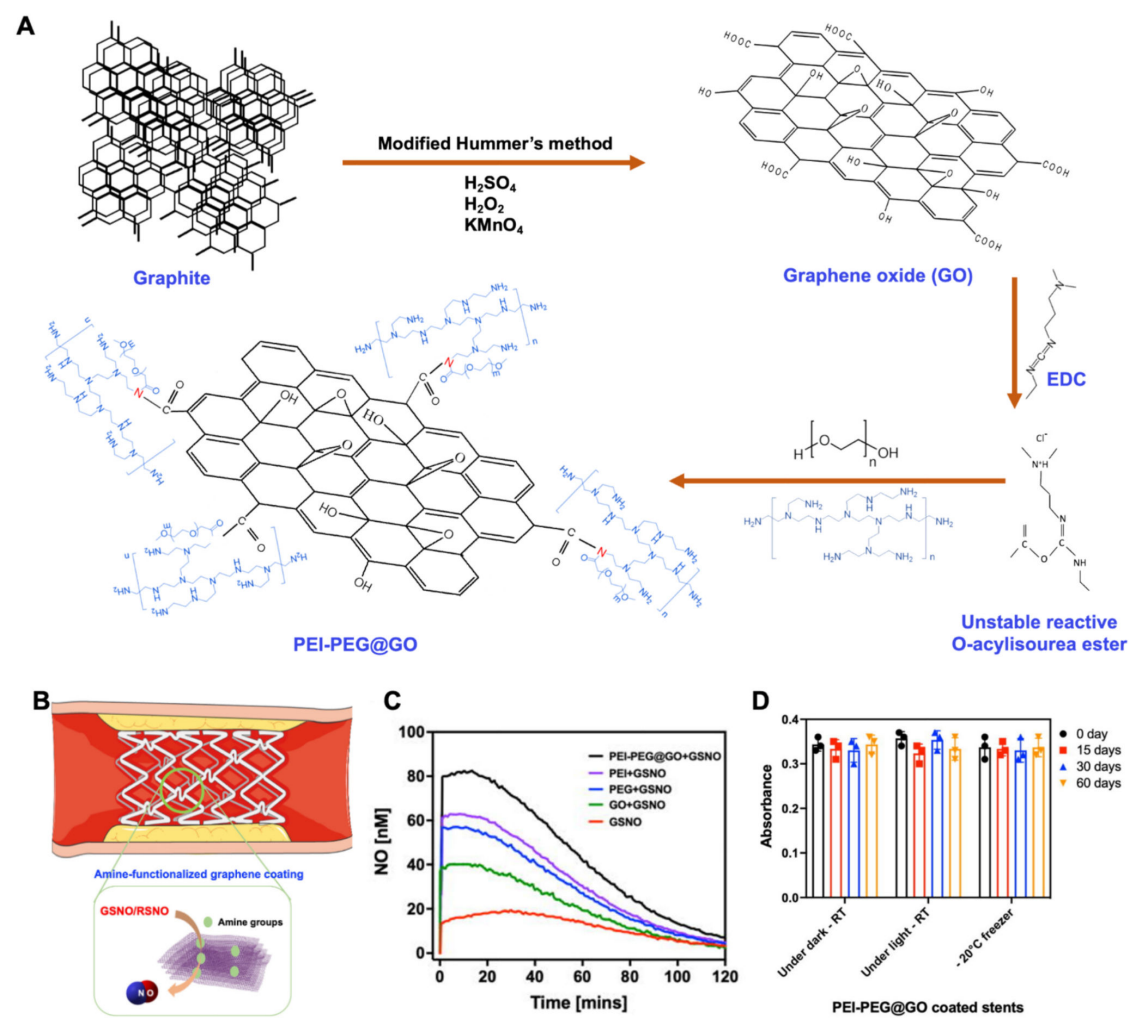
Amine-functionalized GO for the catalytic generation of NO. (A) The design and synthesis of functionalized GO conjugate (termed as PEI-PEG@GO) is schematically represented. GO was prepared by oxidizing graphite flakes using the modified Hummer’s method, then functionalized with PEI and PEG through amide bond formation in the presence of EDC (1-Ethyl-3-(3-dimethylaminopropyl)carbodiimide) as a coupling agent. (B) A schematic illustration of amine-functionalized graphene coating onto a vascular stent. (C) NO release from GSNO was quantified with and without different conjugates using a NO electrode sensor. NO release was measured from GSNO alone (10 μM) and in combination with GO, PEI and PEG, but was highest for the PEI-PEG@GO conjugate (all 250 μg/ml). (D) evaluation of amine group stability on PEI-PEG@GO coated stents for up to 60 days at room temperature (RT). Reproduced with permission from Reference [67]. Abbreviations: PEI: polyethyleneimine; PEG: polyethylene glycol; PEI-PEG@GO: polyethyleneimine-polyethylene glycol functionalized graphene oxide; H_2_SO_4_: sulfuric acid; H_2_O_2_: hydrogen peroxide; KMnO_4_: potassium permanganate; EDC: 1-ethyl-3-(3-dimethylaminopropyl)carbodiimide; GSNO: S-nitrosoglutathione; RSNO: S-nitrosothiol; RT: room temperature.

Our laboratory has synthesized a novel catalyst comprising of polyethyleneimine (PEI) and polyethylene glycol (PEG) covalently bonded to GO (Fig. 4) [67]. PEI was chosen for its rich density of amine groups, which function as nucleophiles due to the lone pair of electrons on the nitrogen atom, which donate electrons and bond with electrophilic species. This facilitates decomposition of the *S*–NO bond to NO under physiological conditions via homolytic and heterolytic cleavage [72]. The multivalent amine groups present in PEI improves its catalytic activity by providing multiple reactive sites for interaction with RSNO, thereby improving both the efficiency and rate of NO generation. PEG was chosen to improve the water dispersibility and colloidal stability of the GO as a catalyst. PEG also improves biocompatibility by minimizing nonspecific protein binding and immune response, which is crucial for both implantable and injectable applications [73]. Furthermore, PEGylation can prolong the circulation time of GO *in vivo* by slowing clearance from the body [74]. The dual conjugation of GO with PEI and PEG results in physiologically stable and catalytically active formulations, making it an ideal candidate for controlled NO delivery in vascular implantable applications. PEI-PEG@GO catalyzed the release of 62 % and 91 % of available NO from GSNO and SNAP respectively and enhanced real-time NO release under physiological conditions. As a proof-of-principle, we demonstrated that amine-functionalized GO could be coated onto biodegradable vascular stents via a simple dipcoating method. This was optimized to achieve a uniform PEI-PEG@GO coating, stable for at least 60 days, while maintaining polylactic acid (PLA) biodegradability during accelerated degradation testing. This innovative approach demonstrates the potential of polymeric amine-functionalized GO as a catalyst to generate physiologically relevant concentrations of NO from RSNOs. Further work is required to determine the capacity of this approach for long-term delivery of NO *in vivo* and to test efficacy in models of vascular injury.

### 4.2 Gas adsorption (physical adsorption)

In recent years, gas storage in systems such as carbon materials and porous materials, including metal organic frameworks (MOFs), have attracted great interest for a wide range of applications including energy, environment and medicine. Similarly, the large theoretical specific surface area of graphene (~2600 m^2^/g), provides a high capacity for storing NO gas. Fluorocarbons also have utility as gas carriers due to high specific surface area and low intermolecular van der Waals interactions, but they exhibit extremely low water dispersibility. Workie et al. [75] describe a composite NO gas delivery system, by non-covalently conjugating fluorinated PEG (chosen to increase hydrophilicity) with acid-treated GO. The F-PEG@GO composite had more vacancies and higher porosity, resulting in a surface area of 87 m^2^/g, compared to 43 m^2^/g for GO alone, which allowed F-PEG@GO to store more NO gas and release NO at a higher rate.

### 4.3 Stimuli-responsive release of NO

**Fig. 5:**
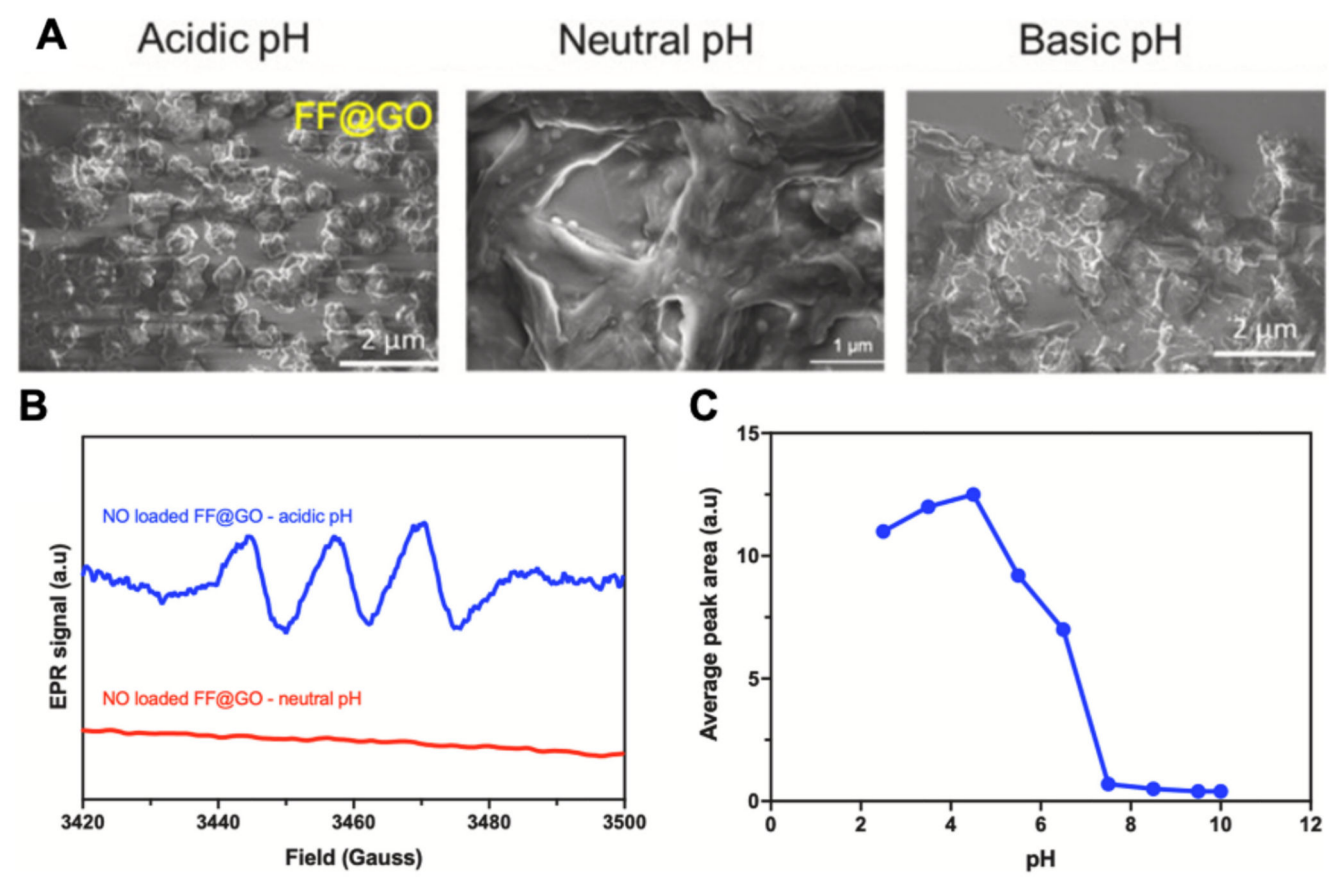
pH-responsive behaviour of a GO hybrid nanosheet. (A) scanning electron microscopy (SEM) images of FF@GO nanosheets demonstrating pH-dependent disruption of morphology under acidic conditions. (B) Release of stored NO gas from FF@GO is detected only at acidic pH, as detected by electron paramagnetic resonance (EPR) using a DETC_2_Fe spin trap. (C) Quantification of NO release by average peak area from EPR spectra as a function of pH. Reproduced with permission from reference [76]. Abbreviations: GO: graphene oxide; FF: diphenylalanine; FF@GO: diphenylalanine peptide-functionalized graphene oxide.

Another design concept for controlled release of NO gas is to make the formulation stimuli responsive. This approach makes use of endogenous triggers, such as altered pH, redox potential, or expression of enzymes, which are characteristic for the target cell type or disease state. Our laboratory developed a novel pH-sensitive NO release system, which was achieved by the co-assembly of hybrid nanosheets consisting of GO wrapped in dipeptide diphenylalanine (FF) via weak molecular interactions including electrostatic, hydrogen bonding and π-π stacking (termed FF@GO) [76]. These interactions are pH sensitive due to the presence of carboxylic acid and amine functionalities on GO and the dipeptide building blocks. We demonstrate that this formulation can be loaded with NO gas, with the dipeptide acting as an arresting agent to inhibit NO burst release at neutral pH; however, at acidic pH it is capable of releasing NO at the rate of up to 0.6 μM per minute. The pH-responsive features of FF@GO are shown in Fig. 5, whereby the morphology of FF@GO nanosheets is preserved at basic and neutral pH, while there is disassembly in an acidic environment. This was confirmed by the detection of NO release only at acidic pH. These findings demonstrate the potential of hybrid GO formulations to achieve delivery of NO specifically to diseased cells, e.g. in response to the acidic environment found in ischaemia.

**Fig. 6:**
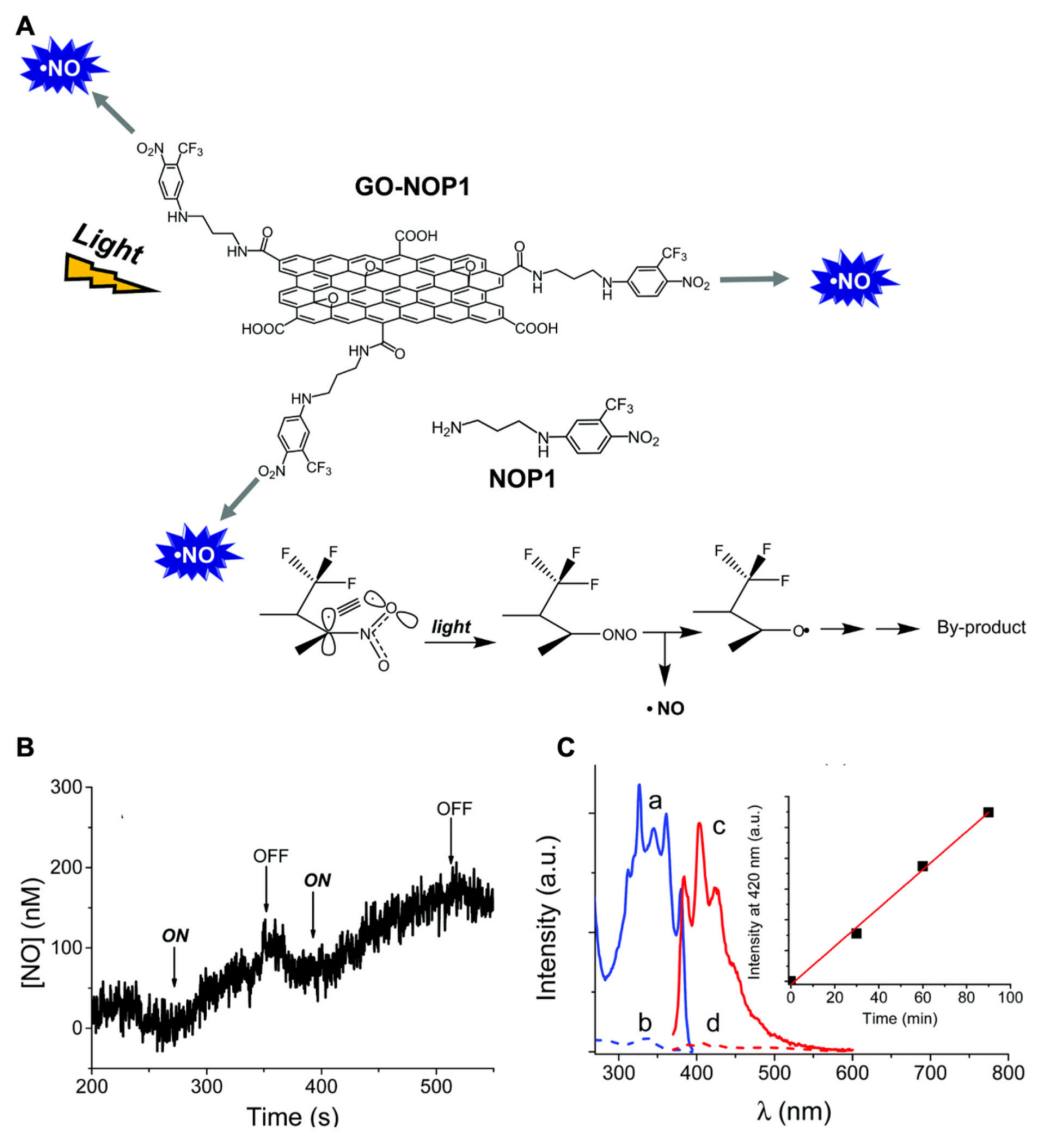
Light-responsive NO release from functionalized GO. (A) Molecular structure of the NO photodonor, NOP1, conjugated to GO to make a photoactivatable GO–NOP1 hybrid, including a schematic representation of the mechanism for photochemical release of NO. (B) NO release profile from a GO–NOP1 aqueous suspension using ON/OFF cycles of UV light at 405 nm. (C) Fluorescence excitation (a, b = 410 nm) and emission (c, d = 360 nm) spectra from a NO-sensitive fluorophore in the presence of GO–NOP1. Minimal fluorescence is detected under dark conditions (dotted line), which is in contrast with 60 min of light exposure at 405 nm (solid line). The inset illustrates fluorescence intensity as a function of light exposure time indicating cumulative NO release. Reproduced from reference [78]. Abbreviations: GO: graphene oxide; NOP1: nitric oxide photodonor 1.

Light can be used as a stimulus for the precise, non-invasive and real-time control of drug release, such that light-responsive materials have the potential to improve efficacy of the therapeutic payload while minimizing side effects [77]. This approach has been employed for NO release using graphene-based materials, for example, Marino et al. [78] developed a light-controlled delivery system by functionalizing GO covalently with an amino-terminated NO photodonor (NOP1) (Fig. 6A), which releases NO upon illumination with UV light (410 nm). They demonstrate that the efficiency of NOP1 photodecomposition is unaffected by integration into the GO scaffold, while avoiding any significant quenching effect (i.e. photoinduced energy/electron transfer). The results illustrated in Fig. 6B provide evidence that GO–NOP1 is stable in the dark but releases NO upon illumination with UV light, as confirmed by repeated ON/OFF cycles of illumination. Photo-release of NO was further confirmed using a fluorometric assay, whereby 2,3-diamino-naphthalene (DAN) reacts with nitrite (a degradation product of NO) to form the fluorescent derivative 2,3-naphthotriazole (DANT) (Fig. 6C). The emission intensity of DANT was shown to increase linearly upon illumination as a result of increasing NO release (inset of Fig. 6C). However, the dependence on UV light may greatly limit clinical applications, since light at this wavelength is strongly absorbed by proteins, nucleic acids and other chromophores, which limits penetration to a depth of 1–2 mm. Light responsive delivery to deeper tissues and organs is much more challenging, as this typically requires systems responsive to near-infrared (NIR) light which offers superior tissue penetration and minimal absorption by biomolecules [79].

### 4.4 Encapsulation of NO donors via covalent bonding

An alternative approach is to facilitate covalent bonds with NO donors by using nanomaterials that are more prone to chemical reactions or by modifying chemical groups on the surface of nanomaterials. Release of NO from the encapsulated donor may be due to spontaneous degradation of the nanomaterial or by exposure to light or ultrasound to break the covalent bonds. NONOates are a class of NO donors that have been extensively studied. They offer the advantage of not requiring catalytic reactions or redox mechanisms for NO release and can be chemically modified with cleavable linkers to achieve cell/tissue-specificity based on enzyme expression levels. Tanum et al. [80] conjugated NONOates with GO to develop a multilayer film via layer-by-layer assembly. They used the intra-H bonding of neighbouring cation amines and intercalated water molecules between GO layers to stabilize the NONOates. The total release of NO from the multilayer GO film was 0.3 ± 1.0 μmol.cm^−2^, with a release duration 15- and 2-times longer than those for the GO solution and the monolayer GO, respectively. Clearly NONOate conjugation with GO is a promising approach for NO delivery, however, concerns remain about the potential for generation of carcinogenic nitrosamines, which are formed by hydrophilic NONOates and amine leaching from the layers of GO film.

Garren et al.[81] developed *S*-nitrosated GO nanosheets via covalent immobilization of SNAP to GO nanosheets which had been functionalized with amine groups for the catalytic release of NO. Our laboratory took a similar approach of developing *S*-nitrosated nanosheets, but introduced thiol groups by conjugation of cysteamine to GO or porous GO (i.e. GO with additional pores on the basal plane), which were then reacted with acidified sodium nitrite to produce *S*-nitrosated formulations [66]. This design represents a straightforward and scalable strategy for larger-scale applications when compared to SNAP-functionalized approaches. NO released from the formulations was sustained at > 5 × 10^−10^ mol cm^−2^ min^−1^ for at least 3 h, which is comparable to NO production by healthy endothelium (0.5–4 × 10^−10^ mol cm^−2^ min^−1^ [39], while production of toxic *N*-nitrosamines was negligible. Furthermore, the porous GO demonstrated significantly higher surface area than GO, allowing for a greater NO payload when compared to the non-porous GO nanosheets used by Garren et al. [81]. In another study, Tian et al. [82] reported on GO loaded with L-arginine, with a surface layer of soybean lecithin to improve the hydrophilicity and biocompatibility of the formulation. L-arginine is the natural endogenous sub-strate for the generation of NO by NOS enzymes, however, this study does not provide direct evidence of NO release from the hybrid formulation, nor does it explain whether proposed NO release occurs via enzymatic (iNOS-mediated) or non-enzymatic (ROS-mediated) pathways. Future studies should focus on quantifying NO release kinetics from L-arginine functionalized materials and validating the proposed molecular pathways.

## 5 Applications of NO-releasing graphene

### 5.1 Vascular diseases

Despite significant advancements in interventional and surgical practice, cardiovascular disease remains a leading cause of morbidity and mortality worldwide [83]. The increasingly ageing population in both developed and developing countries has contributed to a significant rise in cardiovascular disease prevalence. It is clinically established that low NO bioavailability is a driving factor for cardiovascular diseases such as angina, heart failure, hypertension, atherosclerosis and coronary artery disease, primarily as a consequence of impaired endothelial function and reduced vasodilation [84]. Two potential applications of NO-releasing graphene have recently been described by our laboratory, namely, injectables for the prevention of myocardial ischaemia–reperfusion (I/R) injury and as a coating for vascular stents and other medical devices.

Vascular stents are metal mesh tubes commonly used for the treatment of coronary artery disease, whereby blood flow is restored to blocked or narrowed arteries and a stent inserted to maintain vessel patency while the underlying lesion heals [85]. A common complication is in-stent restenosis, i.e. a re-narrowing of the vessel caused by uncontrolled growth of the underlying smooth muscle cells (neointimal hyperplasia), inflammation and thrombosis. For this reason, drug-eluting stents (DES) are conventionally used in the clinic to reduce the incidence of restenosis via the gradual release of potent antiproliferative drugs such as paclitaxel and sirolimus. However, although efficient in reducing neointimal proliferation, these drugs may delay re-endothelialization and increase the risk of late thrombosis [86]. The local release or generation of NO represents an alternative and potentially superior approach, since it not only prevents smooth muscle cell proliferation, but also has vasodilatory, anti-inflammatory and antiplatelet actions, while promoting endothelialisation and vascular healing [87]. However, NO-releasing stents are limited by unstable NO donors, short duration of NO release and uncontrolled release kinetics [9] and we have therefore developed a stent coating consisting of GO functionalized with amine groups to catalytically generate NO from endogenous sources present in the blood stream (as described in Section 4 and in Fig. 4). This has the potential to confer enhanced surface stability, extended NO generation and tuneable release profiles [67], but further work is required to test the efficacy of this approach for long-term delivery of NO *in vivo*.

In another study, we tethered acidified sodium nitrite covalently to the thiol groups of cysteamine-functionalized porous GO [66] (see section 4.4 for formulation details). This formulation significantly promoted the proliferation of endothelial cells and inhibited the proliferation of murine smooth muscle cells (MOVAS) *in vitro*, effects that were associated with the improved release of cyclic GMP (cGMP), thereby confirming intracellular activation of the canonical NO signalling pathway. Single-cell Raman micro-spectroscopy was further used to demonstrate the uptake and localization of porous GO by murine endothelial (sEND.1) and smooth muscle cells (MOVAS), with intracellular NO release detected using a fluorescent NO-specific probe.

### 5.2 Ischaemia / reperfusion injury

Myocardial ischemia–reperfusion (I/R) injury is a common complication of restoring blood flow to the ischaemic heart. The extent of tissue damage is not only dependent on the duration of ischaemia, but is also a consequence of reperfusion with oxygen-rich blood, which interacts with accumulated metabolites to cause mitochondrial dysfunction and oxidative stress [88,89]. Key studies using mitochondria-targeted RSNOs (e.g. mito-SNO) have demonstrated that burst release of exogenous NO during early reperfusion can temporarily inhibit mitochondrial respiration at complex I to prevent ROS production and reduce I/R injury in mice [13]. However, delivering NO to under-perfused tissues during the narrow reperfusion window remains challenging, so approaches that enable rapid, timely and localized NO delivery are highly desirable. For this purpose, we have developed a pH-responsive NO-releasing GO-peptide nanohybrid, designed to release NO gas selectively under the acidic environment found in ischaemic cells (see section 4.3 and Fig. 5 for formulation details) [76]. Proof-of-principle testing using *in vivo* disease models is an important next step.

### 5.3 Antibacterial and antibiofilm applications

Bacterial infections pose a major threat to human health due to their high pathogenicity and associated morbidity and mortality, resulting in a significant economic burden globally [90]. Although antibiotics play a crucial role in combating bacterial infections, the rise of antimicrobial resistance has considerably complicated the situation [91], in particular, methicillin-resistant Staphylococcus aureus (MRSA) and multi-resistant Pseudomonas aeruginosa (MRPA). Developing innovative antimicrobial approaches is imperative and NO emerges as a promising antimicrobial agent due to its broad-spectrum antimicrobial actions based on oxidative and nitrosative damage to microbial DNA, enzymes, proteins and membranes (as explained in Section 1). A variety of NO systems have been developed such as NO gas, acidified nitrite creams, NO donor drugs, RSNOs, NONOates and NO-releasing patches.

**Fig. 7:**
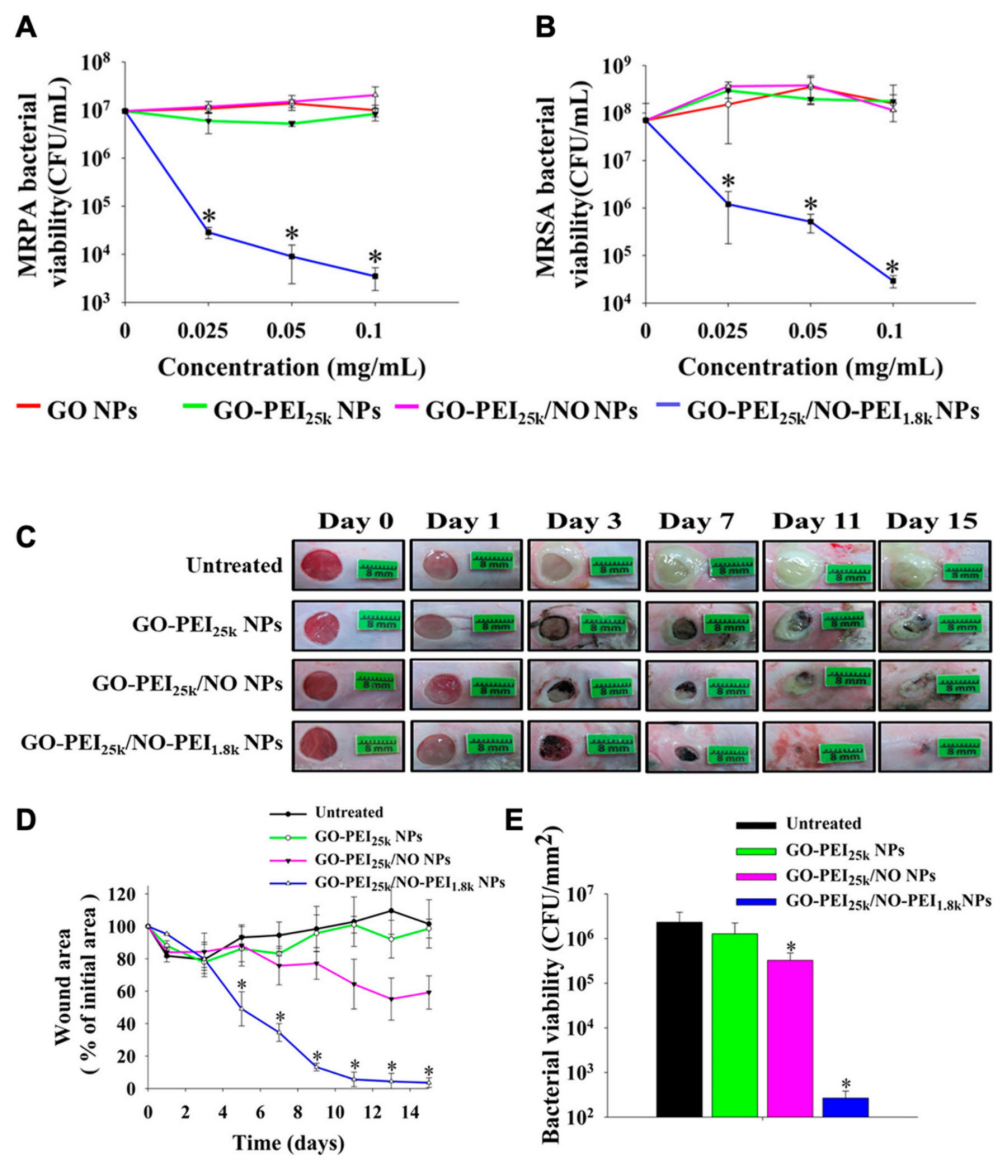
Antibacterial activities of GO, GO-PEI_25k_, GO-PEI_25k_/NO and GO-PEI_25k_/NO-PEI_1.8k_ formulations incubated for 24 h against (A) MRPA and (B) MRSA. (C) Representative images of MRPA-infected wound healing in mice treated with GO-PEI_25k_, GO-PEI_25k_/NO and GO-PEI_25k_/NO-PEI_1.8k_. (D) Area reduction (%) profiles of the MRPA infected wounds. (E) Bacterial burden on the wound (CFU) 15 days postinjury. Reproduced with permission from Ref. [92] Abbreviations: GO NPs: graphene oxide nanoparticles; GO-PEI_25k_: graphene oxide functionalized with polyethyleneimine (25,000 Da); MRPA bacteria: multidrug-resistant Pseudomonas aeruginosa; MRSA bacteria: methicillin-resistant Staphylococcus aureus; CFU/mL: colony-forming units per milliliter.

In a study by Cao et al. [92], GO was functionalized with PEI and loaded with NO gas (GO-PEI_25k_/NO-PEI_1.8 k_). Colony-forming unit (CFU) assays demonstrated robust antimicrobial effects in comparison to unmodified GO, achieving 4 log units of bacterial (MRPA and MRSA) reduction at 0.1 mg/ml concentration within 8 h (Fig. 7 A and B). The presence of cationic PEI improved bacterial adhesion and localized NO release at the bacterial surface, thereby disrupting bacterial membranes. Efficacy was confirmed *in vivo* in mice, where nanoparticles of GO-PEI_25k_/NO-PEI_1.8 k_ significantly reduced the size of MRPA-infected wounds by 50 % within 5 days and achieved nearly 90 % wound contraction after 9 days. In contrast, the untreated group and NO-free formulations demonstrated minimal wound healing (Fig. 7 C, D and E). Histological studies indicated that GO-PEI_25k_/NO-PEI_1.8 k_ acted to support re-epithelialization, improved collagen deposition and reduced chronic inflammation, resulting in tissue regeneration resembling normal skin. This study demonstrates the promise of NO release from PEI-conjugated GO for enhanced antimicrobial activity and accelerating wound repair even in the presence of drug-resistant bacteria.

Biofilm formation significantly reduces bacterial susceptibility to antibiotics. It has been established that the antibiotic concentration needed to eradicate bacteria within biofilms is approximately 1,000 times higher compared to that required for independently living bacteria [93]. For example, NO-releasing GO nanosheets, developed by Garren et al. [81] via surface aminosilylation and thiolation, exhibited superior antibacterial and antibiofilm properties compared to unmodified GO. Amine groups were introduced to GO through (3-aminopropyl)trie-thoxysilane (APTES) modification, followed by the incorporation of thiol groups via thiolactone aminolysis and finally, the modified GO was nitrosated with nitrite to fabricate NO releasing GO formulation. The functionalized GO nanosheets significantly reduced *E. coli* and MRSA adhesion and biofilm biomass by up to 60 % and 42 %, respectively after 24 h, compared to ~ 25 % and 17 % reductions for unmodified GO. This was attributed to a combination of NO release and the surface charge disrupting bacterial membranes. These results suggest the potential of NO-releasing graphene formulations as coatings on medical devices, such as indwelling catheters, to prevent clinical complications that arise as a consequence of biofilm formation.

In recent years, there has been growing interest in developing combination therapies, which combine NO release with other agents for improved therapeutic outcomes. For example, photothermal therapy (PTT) utilizes light-sensitive compounds to convert absorbed light energy into heat, which can kill bacteria and disrupt biofilm formation [94–96]. Although PTT has shown promising antimicrobial results, the high dose of photothermal agents and/or high temperature may induce unwanted cytotoxicity [97,98]. The superior photothermal conversion efficiency of graphene-based materials combined with the ability to functionalize with NO-releasing capabilities, make them promising candidates for developing a synergistic dual-mode strategy. To further improve localized NO release, researchers have explored the integration of graphene into advanced delivery platforms, such as hydrogels and microneedles. Hydrogels mimic the extracellular matrix to support tissue regeneration and healing, while graphene-integrated microneedles improve drug penetration into infected tissues, disrupt biofilms and deliver targeted payloads with minimal pain and systemic toxicity.

He et al. [99] developed multifunctional hydrogels by incorporating graphene-based materials into a polymeric network and by combining NO-release and photothermal effects. The hydrogel-graphene composite exhibited 100 % bacterial killing for *S. aureus* and *E. coli* within 10 min. The composite achieved complete wound closure in diabetic mice within 21 days, while promoting angiogenesis and tissue regeneration in comparison to commercially available dressings. Ma et al. [100] introduced hydrogel microneedles incorporated with GO and the NO donor, GSNO. Under near infra-red (NIR) irradiation, these formulations reduced MRSA biofilm forming bacteria by 3-log and effectively disrupted biofilm formation. *In vivo* results revealed accelerated wound healing and superior collagen formation, highlighting their promise as a minimally invasive approach for wound healing. Huang et al. [101] reported a β-cyclodextrin-functionalized GO hydrogel conjugated with the NO donor BNN6. Under NIR irradiation, the hydrogel composite exhibited synergistic bacterial inactivation rates of 97.6 % and 95.5 % for *S. aureus* and *E. coli*, respectively. Application to a bacteria-infected wound model *in vivo*, demonstrated near complete re-epithelialization and collagen formation within 14 days, while reducing inflammation and stimulating angiogenesis. These studies are highly promising; however, further work to determine the exact mechanisms of action of NO and its by-products could prove useful for the rational optimisation of formulations.

### 5.4 Anti-cancer

Cancer remains the second leading cause of death globally [102] despite conventional treatment modalities, including chemotherapy, surgery and radiotherapy [103]. Surgery typically fails to remove small residual tumors and tumor cells spread across blood vessels and lymphatics, promoting recurrence and metastasis [104], while chemotherapy is often compromised by the emergence of drug-resistant cancer cells. In addition, the hypoxic nature of tumors significantly reduces the efficacy of radiotherapy [105]. New therapies including phototherapy (PTT, photodynamic therapy), gas therapy and immunotherapy offer advantages over conventional treatments [106,107]. This section covers recent advances in the development of NO-based graphene for cancer treatment including in combination with other therapies.

Combining NO release with PTT has shown promise to overcome tumor resistance. PTT utilizes NIR-induced hyperthermia to kill tumor cells and the addition of NO can improve therapeutic efficacy. Mechanistically, NO disrupts cellular signalling, induces intracellular oxidative stress and inhibits mitochondrial respiration, which sensitize tumor cells to thermal damage and heat-triggered apoptosis [108–110]. Two recent studies exemplify the potential for synergy when combining precise NO delivery with photothermal effects. Tian et al. [82] reported a GO-L-arginine formulation functionalized with soybean lecithin to improve the biocompatibility and stability of formulations. NIR irradiation at 808 nm increased tumor temperatures to 47.5 °C within 5 min. In a 4T1 breast tumor model in mice, intravenous injection of the formulation eradicated tumors, suppressed recurrence and activated cytotoxic T lymphocytes. In a similar fashion, Guo et al. [111] developed a nanosystem of nitrogen-doped graphene quantum dots, using ruthenium nitrosyl as a NO donor and a triphenylphosphonium (TPP^+^) moiety for mitochondria targeting. Upon 808 nm NIR irradiation, this nanosystem released NO instantaneously and achieved significant hyperthermia, with tumor site temperatures increasing by 16.7 °C. The ruthenium-based NO donor provided precise spatiotemporal control over NO release specifically to mitochondria of human cervical carcinoma cells (HeLa cells), enhancing the cytotoxic responses. In HeLa tumor-bearing mice, the nanosystem achieved significant tumor regression and eradication. Together, these studies exemplify the versatility of NO-releasing graphene platforms, combining both PTT and NO delivery to target tumors through synergistic mechanisms. However, limited light penetration and excessive or prolonged hyperthermia may adversely affect surrounding healthy tissues. To achieve an optimal balance between anticancer efficacy and safety, factors such as NO release kinetics, cytotoxicity and light penetration depth (particularly for deep tumors) and accurate temperature and heat exposure/regulation must be finetuned by manipulating size, shape, light-responsiveness, aggregation state and surface chemistry.

## 6 Comparison of NO delivery nanosystems

### 6.1 Alternative approaches

Multiple nanoparticle systems have been explored for NO delivery applications, however, so far, several limitations have hampered their translation to clinical use. For example, polymeric nanoparticles, lipid nanoparticles and hydrogels typically suffer from low NO loading efficiencies and uncontrolled burst release kinetics, which can result in suboptimal dosing and limited therapeutic duration. Lipid nanoparticles while inherently biocompatible, aggregate and degrade rapidly without ultra-cold storage conditions (e.g. – 80 °C as for mRNA vaccines) [113], which limits their broader applicability, particularly in long-term and implantable applications. Although lipid-based nanoparticles are highly efficient for nucleic acid delivery, they are less suitable for small hydrophobic molecules and therapeutic gases due to limited encapsulation capacity. The lack of mechanical strength further restricts their use for applications requiring structural and mechanical support such as vascular stents [114]. Hydrogels offer tunable structural features, but their NO delivery applications suffer from leaching, swelling, weak mechanical integrity and variable biocompatibility [115,116]. Swelling may deform the shape and porosity of a device and reduce effectiveness in physiological environments. Metal–organic frameworks (MOFs) represent a promising class of porous carriers due to their tunable porosity, high specific surface area and exceptional gas-loading capabilities. MOFs such as MIP-177 and MIP-210 exhibit excellent NO adsorption [117]; however, many MOFs such as MIL-100 and MIL-127 show poor stability in biological fluids [118]. They degrade rapidly in phosphate-rich biological environments, leading to premature gas release. Also, biodegradation of MOFs may release toxic metal ions upon dissociation of the metal–ligand coordination bonds, raising toxicity and safety concerns [119].

Collectively, this highlights the challenges of targeted NO delivery for clinical use, which requires the development of nanocarriers with high loading capacity, targeted release and improved *in vivo* stability. In response, drug carrier design is becoming more sophisticated, incorporating targeted delivery mechanisms and prodrug strategies to improve therapeutic precision.

### 6.2 Advantages of graphene

A fundamental advantage of graphene nanomaterials lies in their exceptionally high specific surface area (~2600 m^2^/g), allowing for superior loading of NO donors or catalytic compounds compared to conventional nanomaterials. Furthermore, the remarkable mechanical strength (~200 GPa) and non-swelling stable interlayer nanochannels and tunable surface chemistry provide an ideal platform for incorporating a variety of NO donor molecules (e.g., RSNOs, NONOates, nitrites, NO gas), catalysts (metal ions, nucleophiles) and reducing agents or triggers (heat, ultraviolet or near-infrared light) for finely controlled, sustained and tunable NO release. Graphene-based coating onto implants can maintain their structural integrity and withstand dynamic mechanical stress under physiological conditions. Graphene oxide (GO)-based formulations can be engineered to respond to multiple stimuli both endogenous and exogenous including changes in pH, light exposure, redox changes and enzymatic activity, allowing precise spatial, temporal and concentration dependent control over NO release.

### 6.3 Clinical translation

Functionalized GO has demonstrated tremendous promise, with low toxicity in therapeutically relevant concentrations, but data on *in vivo* toxicity remains sparse. In a landmark study, the Kostarelos group recently conducted a first-in-human randomized controlled trial to evaluate the acute pulmonary and cardiovascular safety of inhaled GO nanosheets in 14 healthy volunteers [120]. The lungs were chosen due to their critical role in filtering inhaled substances and as a model organ with exquisite and measurable sensitivity. The study found no significant changes in heart rate, blood pressure, lung function or systemic inflammation, demonstrating the short-term tolerability of GO exposure under clinically controlled conditions. In parallel, the same group is leading a first-in-human clinical investigation [121] of graphene-based brain-computer interfaces in patients undergoing neurosurgery, which could open new avenues for future neuromodulation and diagnostics applications. Collectively, these foundational studies highlight the translational potential of graphene and provide encouraging data on safety for human use, but clearly much more remains to be done.

## 7 Conclusion and future perspectives

In this review we have summarized advancements in the development of NO-releasing graphene for applications in cardiovascular diseases, antimicrobial therapy and cancer treatment. NO-releasing graphene is emerging as a next-generation therapeutic platform owing to several key advantages over other NO delivery nanosystems including high specific surface area which allows for superior NO loading efficiency and ease of functionalization with NO donors or catalytic agents as well as its robust mechanical strength and chemical stability. The studies discussed herein demonstrate the promise of this platform, however, significant limitations remain unaddressed, including a lack of *in vivo* data for cardiovascular diseases and limited mechanistic insights for infections and cancer. That said, many of these limitations are not unique to NO-releasing graphene but are also common across the field of NO-releasing nanoparticles.

For example, the rapid diffusion and short half-life of NO remains a major challenge, which makes precise targeting difficult and leads to undesirable toxic effects. Recent work on stimuli-responsive delivery systems represents an important advance in this regard, since it can release NO at sites of disease in response to local cues, such as, pH, redox potential and enzyme expression levels. Other *active* targeting approaches could be used to direct delivery systems to particular cell types by conjugating antibodies, aptamers or ligands, which are recognised by specific receptors and biomolecules, and graphene is particularly well suited to this type of conjugation.

The tunable surface chemistry of graphene-based nanomaterials can also enable controlled and stimuli-responsive NO release in response to external triggers, particularly via light. The inherent photothermal properties of graphene can be exploited to synergistically trigger NO release via localized heating. For example, the combination of photothermal therapy (PTT) with NO release has demonstrated remarkable improvements in bacterial killing and biofilm disruption and illustrates the potential for employing multi-pronged, synergistic, approaches. It is worth noting that the same graphene nanotechnology can also be used to deliver therapeutic payloads such as nucleic acids, siRNA, DNA, peptides, proteins and antibodies, either alone or in combination with NO release.

Despite promising early results, research on NO delivery using graphene remains limited, partly due to the multidisciplinary expertise required to address challenges in materials science, biology and medicine. Future research challenges include detecting and understanding the physiological relevance of NO metabolites, particularly those by-products with potential for toxicity. Oral administration of NO-releasing graphene presents further barriers, in terms of absorption and surviving first-pass metabolism by the liver.

In addition, there are outstanding questions concerning the entry, clearance and excretion pathways for graphene. For example, what are the pathways for intracellular trafficking of graphene in target cells? What is the nature of interactions between graphene with healthy cells and blood components? How does graphene enter the cell and where does it end up? What are the degradation products of graphene and how are they cleared from the body? *In vivo* studies must assess the biological fate of graphene, as well as the fate of degraded graphene particles within the living system. Understanding the long-term impact on biological systems will be critical for developing safe and effective applications. There remains a translational gap between preclinical development and progression to clinical trials.

In conclusion, while NO-releasing graphene shows great promise, its journey toward clinical application requires concerted efforts across disciplines to overcome technical, mechanistic and safety challenges, only then will we realise the potential of graphene as a transformative delivery platform in medicine.

## Figures and Tables

**Table 1:** Graphene based formulations for the therapeutic delivery of nitric oxide.

Graphene carrier	NO source	Formulation design	NO generation and release trigger	Disease model and outcomes
Porous graphene oxide [66]	*S*-nitroso-cysteamine	*S*-nitrosated nanosheets prepared via cysteamine thiolation followed by sodium nitrite-mediated nitrosation	>5 × 10^−10^ mol cm^−2^ min^−1^ for 3 h via thiol catalyzed S-NO bond cleavage	*In vitro:* increased endothelial proliferation inhibited smooth muscle cell proliferation activation of canonical NO signalling Applications: cardiovascular diseases (CVD), vascular injury
Graphene oxide [76]	NO gas	Self-assembling GO/diphenylalanine hybrid nanosheets via non-covalent interactions	Up to 0.6 pM per minute Acidic pH to release NO	pH-responsive NO release (cell-free) Applications: ischaemic diseases; ischaemia/reperfusion injury
Graphene oxide [67]	Endogenous RSNO	PEI — functionalized for the decomposition of *S*-nitrosothiols to release NO. PEGylated to improve biocompatibility	62 % NO release from GSNO and 91 % from SNAP. Spontaneous decomposition.	NO release from *S*-nitrosothiols (cell-free). Stabile coating on bioabsorbable stents. Applications: coating for vascular stents or implantable devices
Graphene oxide[99]	Thermo-responsive NO donor (BNN6)	A composite of carboxymethyl chitosan (a biopolymer), 2,3,4-trihydroxybenzaldehyde, copper chloride, GO and BNN6	Up to 3 μM over 10 min triggered by near-infrared (NIR) laser irradiation	Antibacterial against *S. aureus* and *E. coli*. HUVECs: improved proliferation, migration and tube formation, promoting angiogenesis for wound healing. *In vivo*: accelerated wound contraction, collagen content and angiogenesis. Disease model: type 1 diabetic mice Applications: Diabetic wound healing
Graphene oxide [101]	Thermo-responsive NO donor (BNN6)	β-cyclodextrin-functionalized GO loaded with BNN6 for near-infrared light-triggered NO release	4 uM up to 20 min with NIR 2.5 uM up to 20 min without NIR	Antibacterial against *S. aureus* and *E. coli*. cell viability. *In vivo*: Accelerated wound healing, reduced inflammation, improved collagen deposition and promoted angiogenesis. *S. aureus* — infected wound model in male Sprague Dawley rats Applications: Bacteria-infected wound healing.
Graphene oxide [81]	*S*-nitrosothiol	GO-(NH)_x_-SH nitrosated under acidified nitrite conditions	Total: 80 uM over 60 min *S*-nitrosation	High cytocompatibility (fibroblasts) Antibacterial against *S. aureus* and *E. coli*, with significant reductions in bacterial adhesion (up to 92 %) and biofilm mass (up to 60 %). Applications: antibacterial and antibiofilm coatings for medical devices.
Graphene Oxide [92]	NO (g) at 80 psi for 3 days at room temperature	Graphene oxide functionalized with PEI (25 kDa)	400–600 nM per minute up to 40 h.	Low cytotoxicity (L929 mouse fibroblasts) Antibacterial against MRPA and MRSA *In vivo:* Male ICR mice — Improved antibacterial activity, reepithelialization and collagen deposition. Application: MRPA-infected wound healing
Graphene oxide [100]	*S*-nitrosoglutathione	Hydrogel-forming microneedle (HFMN) combining polyvinyl alcohol (PVA) hydrogels with GSNO and graphene oxide	15–32 nM/mg per minute over 25 h with 20 % GSNO loading triggered by IR light	Antibacterial against MRSA (GDMCC 1.771) and *Pseudomonas aeruginosa*. *In vivo*: Female C57BL/6 mice. Reduced bacterial diversity in biofilm wounds Applications: Biofilm-infected wound healing
Graphene oxide [78]	Amino-terminated NO photodonor (NOP1)		25–200 nM per sec over 550 s. Wavelength: 410 nm	Formulations remain stable in the dark and can supply NO under visible light exposure.
Graphene oxide [112]	Generation of nitroxyl from physiologically abundant glucose and L-arginine	Graphene–haemin–glucose oxidase catalysts	0.001 – 0.6 pM per minute, over 20 min catalyzed by endogenous sources.	*In vivo:* coating effectively prevented blood clot formation upon exposure to arterial blood in New Zealand white rabbits. Application: Antithrombotic coating for blood-contacting devices.
Graphene oxide [75]	NO gas	Fluorinated PEG noncovalently conjugated with acid-treated GO	Initial release of ~50 mM/g per min, decreasing to ~5 mM/g per min over 180 min. Adsorption/desorption mechanims	Antibacterial against *E. coli (Gram-negative) and S. aureus (Gram-positive) strain*
Graphene oxide [80]	N-diazeniumdiolate (NONOate)	GO-based multilayer films conjugated with polyethylene terephthalateglycol	Up to 0.27 mM over 70 h	Application: antibacterial. Human dermal fibroblasts (HDFs): inhibition of cell proliferation
Graphene quantum dots [111]	Ruthenium nitrosyl	N-doped graphene quantum dots functionalized with triphenylphosphonium for mitochondria targeting		Application: potential use in wound healing Human cervical carcinoma cells (HeLa cells): mitochondria targeting, induced apoptosis and improved cytotoxicity. *In vivo:* tumor bearing mice. Effective anti- tumor efficacy via NO + PTT synergy
Graphene oxide [82]	L-arginine	L-arginine and soybean lecithin functionalized hybrid GO	NIR laser	Application: cancer therapy 4 T1 (breast tumor cell line) and A549 (lung adenocarcinoma cell line): inhibited tumor growth and recurrence via NO + PTT synergy *In vivo:* 4T1 tumor bearing mice showed excellent photothermal conversion and iNOS mediated NO release in tumor microenvironment Application: cancer therapy
